# Increased Plasma Citrulline in Mice Marks Diet-Induced Obesity and May Predict the Development of the Metabolic Syndrome

**DOI:** 10.1371/journal.pone.0063950

**Published:** 2013-05-14

**Authors:** Manuela Sailer, Christoph Dahlhoff, Pieter Giesbertz, Mena K. Eidens, Nicole de Wit, Isabel Rubio-Aliaga, Mark V. Boekschoten, Michael Müller, Hannelore Daniel

**Affiliations:** 1 Molecular Nutrition Unit, Research Center for Nutrition and Food Sciences, Technische Universität München, Freising-Weihenstephan, Germany; 2 PhD Graduate School ‘Epigenetics, Imprinting and Nutrition’, Research Center for Nutrition and Food Sciences, Technische Universität München, Freising-Weihenstephan, Germany; 3 Netherlands Nutrigenomics Centre, TI Food & Nutrition, Wageningen University, Wageningen, The Netherlands; 4 Division of Human Nutrition, Wageningen University, Wageningen, The Netherlands; INRA, France

## Abstract

In humans, plasma amino acid concentrations of branched-chain amino acids (BCAA) and aromatic amino acids (AAA) increase in states of obesity, insulin resistance and diabetes. We here assessed whether these putative biomarkers can also be identified in two different obesity and diabetic mouse models. C57BL/6 mice with diet-induced obesity (DIO) mimic the metabolic impairments of obesity in humans characterized by hyperglycemia, hyperinsulinemia and hepatic triglyceride accumulation. Mice treated with streptozotocin (STZ) to induce insulin deficiency were used as a type 1 diabetes model. Plasma amino acid profiling of two high fat (HF) feeding trials revealed that citrulline and ornithine concentrations are elevated in obese mice, while systemic arginine bioavailability (ratio of plasma arginine to ornithine + citrulline) is reduced. In skeletal muscle, HF feeding induced a reduction of arginine levels while citrulline levels were elevated. However, arginine or citrulline remained unchanged in their key metabolic organs, intestine and kidney. Moreover, the intestinal conversion of labeled arginine to ornithine and citrulline *in vitro* remained unaffected by HF feeding excluding the intestine as prime site of these alterations. In liver, citrulline is mainly derived from ornithine in the urea cycle and DIO mice displayed reduced hepatic ornithine levels. Since both amino acids share an antiport mechanism for mitochondrial import and export, elevated plasma citrulline may indicate impaired hepatic amino acid handling in DIO mice. In the insulin deficient mice, plasma citrulline and ornithine levels also increased and additionally these animals displayed elevated BCAA and AAA levels like insulin resistant and diabetic patients. Therefore, type 1 diabetic mice but not DIO mice show the “diabetic fingerprint” of plasma amino acid changes observed in humans. Additionally, citrulline may serve as an early indicator of the obesity-dependent metabolic impairments.

## Introduction

The metabolic syndrome is characterized by central obesity, hyperglycemia, dyslipidemia and insulin resistance (IR) associated with an increased risk for the development of cardiovascular disease and type 2 diabetes mellitus (T2DM). A blunted response of peripheral tissues to insulin action leads to disturbances of carbohydrate, amino acid, and lipid metabolism. Insulin-mediated glucose uptake into skeletal muscle and adipose tissue is impaired and the subsequent hyperglycemia is considered to further promote IR and T2DM. Reduced insulin-mediated suppression of lipolysis and lipid accumulation in non-adipose tissue plays a pivotal role in the development of IR. Insulin also regulates protein biosynthesis in a tissue-specific manner [1] and activates amino acid transporters in skeletal muscle [2]–[4]. Dietary amino acids can stimulate secretion of the incretins gastric inhibitory polypeptide and glucagon-like peptide-1 from intestinal endocrine cells, which further enhance glucose-mediated insulin release from pancreatic β-cells [5], [6]. Selected amino acids can also directly elicit insulin secretion [7], [8]. By activating the mammalian target of rapamycin/raptor pathway, amino acids stimulate S6 kinase 1 leading to an inhibition of insulin signaling by a negative feedback [9]–[11] and IR in turn also affects protein metabolism [12], [13]. Thus, it is not surprising that recent metabolomics studies in obese and diabetic subjects report significant alterations in plasma amino acid levels, particularly elevations of BCAA and AAA [14], [15]. These data confirm essentially the pioneering work of Felig *et al.* who showed increased plasma levels of valine, leucine, isoleucine, tyrosine, and phenylalanine in obese subjects [16]. Similar changes in plasma BCAA are found in Zucker diabetic fatty rats [17], in leptin-deficient ob/ob mice [18], in AKITA mice as a model for type 1 diabetes [19], and in type 1 diabetic patients [20].

DIO in mice is frequently used as a model for metabolic impairments in humans characterized by hyperglycemia, hyperinsulinemia and non-alcoholic fatty liver disease. To assess whether such mice produce the same changes in the plasma amino acid profile as found in obese humans and in subjects with IR and T2DM, we analyzed plasma and tissue samples from C57BL/6 mice fed HF diets for 12 weeks. These mice developed hyperglycemia and showed increased hepatic triglyceride levels [21], [22]. Amino acids and derivatives were quantified using a targeted liquid chromatography-tandem mass spectroscopy (LC-MS/MS) approach with iTRAQ-labeling. We found that mice with DIO possess increased plasma citrulline and ornithine levels compared to control (C) animals. Similar increases of these amino acids were also registered in C57BL/6 mice with insulin-deficiency induced by STZ treatment. In addition, STZ-treated mice, but not DIO mice displayed the expected increases of BCAA and AAA levels in plasma.

## Results

### HF feeding induced changes in plasma

**Figure 1:**
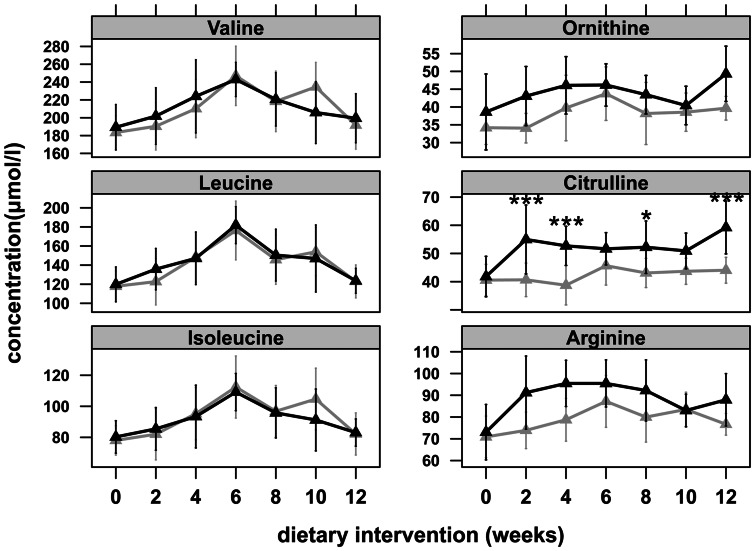
Time-courses of selected plasma amino acid concentrations during 12 weeks of HF dietary intervention in study 1. Grey and black lines represent C (n = 12) and HF animals (n = 12) feeding either a 10 energy% C diet or a 45 energy% HF diet for 12 weeks, respectively. Mice were fasted for 5 hours before they were sacrificed. Data are presented as mean ± SD. Asterisks indicate statistical significance based on two-way ANOVA followed by pairwise comparison using Tukey test. *p<0.05, ***p<0.001.

**Figure 2:**
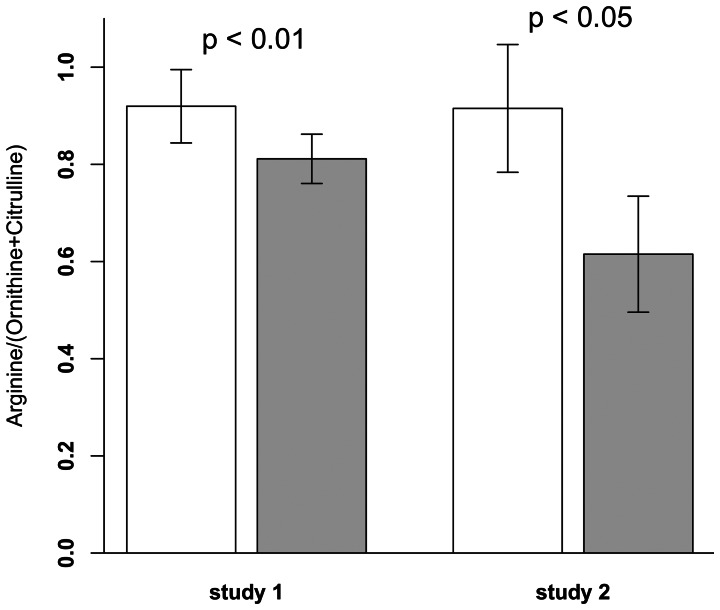
HF intervention reduced systemic arginine bioavailability obtained by the ratio of plasma arginine to ornithine + citrulline. In study 1, mice (n = 12 per group) were either fed a C diet (10 energy% of fat) or a HF diet (45 energy% of fat). In study 2, mice were either fed a C diet (11 energy% of fat, n = 9) or a HF diet (60 energy% of fat, n = 8). After 12 weeks of dietary intervention, mice were sacrificied. Data are presented as mean ± SD. Open bars represent C mice, grey bar represent HF mice. *P*-value obtained by unpaired Student's t-test.

Animals receiving the HF diets gained significantly more weight, displayed hyperglycemia and accumulated more hepatic triglycerides as compared to C animals (Table 1). Whereas plasma concentrations of BCAA and phenylalanine increase in human obesity and T2DM, levels remained unaltered in obese mice and tyrosine levels were significantly elevated only in study 1 (Table 2). Although we observed changes over time, when plasma amino acid concentrations were monitored every second week in study 1, BCAA, phenylalanine, and tyrosine displayed no time x diet interaction based on two-way ANOVA as shown for the BCAA (Figure 1). However, all animals on HF diets displayed increased plasma citrulline and ornithine concentrations after 12 weeks (Table 2) and significant cross-correlations (study 1: r = 0.7, *p*<0.001; study 2: r = 0.68, *p* = 0.004) between citrulline and ornithine were observed. Moreover, as depicted in Figure 1, citrulline also showed a significant two-way interaction for time×diet (*p = *0.002) in study 1. In both feeding trials, the changes in citrulline and ornithine concentrations resulted in a diminished systemic arginine bioavailability [23], [24] defined by the ratio of plasma arginine to ornithine and citrulline levels (Figure 2) while arginine levels were increased in HF animals of study 1 (Table 2).

**Table 1:** Phenotypic parameters of DIO mice after 12 weeks of HF feeding and of STZ-induced diabetic mice.

Study	Parameter	Change	C	HF/STZ	*p*-value
**study 1**	**Body weight (g)**	↑	28.53±1.53	38.74±2.49	<0.001
	**Blood glucose (mM)**	↑	10.39±1.05	13.13±0.65	<0.001
	**Liver triglycerides (mg/g protein)**	↑	363.92±103.37	635.94±191.21	<0.001
**study 2**	**Body weight (g)**	↑	32.14±1.83	42.48±4.32	<0.001
	**Blood glucose (mM)** $	↑	7.60±0.70	9.53±0.86	<0.001
	**Liver triglycerides (mg/g protein)**	↑	221.93±40.33	707.98±251.94	<0.001
**STZ study**	**Body weight (g)**	↓	28.01±2.76	23.22±1.99	<0.001
	**Blood glucose (mM)**	↑	6.06±0.50	23.80±3.60	<0.001
	**Liver triglycerides (mg/g protein)**	-	ND	ND	-

In study 1, mice (n = 12 per group) were either fed a C diet (10 energy% of fat) or a HF diet (45 energy% of fat) for 12 weeks. In study 2, mice were either fed a C diet (11 energy% of fat, n = 9) or a HF diet (60 energy% of fat, n = 8) for 12 weeks. For the STZ-study, parameters were measured five days after mice (n = 10 per group) received a single intraperitoneal injection of STZ (180 mg/kg bodyweight) in 0.1 M citrate buffer or 0.1 M citrate buffer alone (C group).

Data are presented as mean ± SD. *P-*value obtained by unpaired Student's t-test.

$ measured after 11 weeks of HF feeding. ND: not determined.

**Table 2:** Fold-changes of selected plasma amino acids in DIO mice after 12 weeks of HF feeding and STZ-induced diabetic mice.

	study 1		study 2		STZ study	
Amino acid	FC	*p*-value	FC	*p*-value	FC	*p*-value
**Valine**	1.04	0.498	0.9	0.205	1.65	0.007
**Leucine**	1.01	0.916	0.91	0.309	1.71	0.004
**Isoleucine**	1.01	0.848	0.95	0.510	1.58	0.006
**Arginine**	1.15	0.007	0.93	0.419	0.92	0.507
**Citrulline**	1.34	<0.001	1.27	0.015	1.39	0.010
**Ornithine**	1.24	<0.001	1.50	0.004	1.84	0.011
**α-aminobutyrate**	0.85	0.252	0.80	0.079	2.97	0.015
**Tyrosine**	1.23	0.009	1.06	0.557	1.46	0.026
**Phenylalanine**	1.03	0.423	0.96	0.624	1.52	0.020
**Sum**	1.05	0.166	1.09	0.127	1.32	0.029

In study 1, mice (n = 12 per group) were either fed a C diet (10 energy% of fat) or a HF diet (45 energy% of fat) for 12 weeks. In study 2, mice were either fed a C diet (11 energy% of fat, n = 9) or a HF diet (60 energy% of fat, n = 7) for 12 weeks. For the STZ-study, parameters were measured five days after mice (n = 10 per group) received a single intraperitoneal injection of STZ (180 mg/kg bodyweight) in 0.1 M citrate buffer or 0.1 M citrate buffer alone (C group).

FC: fold-change (HF group/C group or STZ group/C group). *P-*value obtained by unpaired Student's t-test.

In addition, analysis of plasma amino acids in HF animals revealed reduced levels of α-aminoadipate in both studies and of hydroxyproline in study 1. Furthermore, increased plasma levels were found for ethanolamine and glutamine in study 1 and for asparagine, serine, and 3-methyl-histidine in study 2. Yet, irrespective of the different dietary fat content (45 energy% in study 1 and 60 energy% in study 2) and fat source (palm oil in study 1 and soybean oil and beef tallow in study 2), the majority of plasma amino acid levels (around 70% in study 1 and 79% in study 2) remained unchanged when compared to lean C mice (data provided in Table S1 and S2).

### Citrulline and ornithine in small intestine and kidney

A major source of systemic citrulline is the intestine. Here it can be produced from either arginine or glutamine. Previous transcriptome profiling of small intestinal tissue samples from study 1 [25] revealed that the mRNA levels of carbamoyl-phosphate synthetase 1 and arginase type II increased significantly (2.2-fold and 1.24-fold, respectively) in animals on HF diets. In particular the increase in arginase type II suggested that intestinal ornithine production from arginine could be increased. In assessing whether the intestine indeed contributes to the increased systemic ornithine and citrulline levels, we employed the everted gut sac technique with jejunal tissues of mice after 12 weeks of feeding C or HF diets and determined the conversion rate of ^13^C^15^N-arginine provided in the luminal solution to either ornithine or citrulline by LC-MS/MS analysis. However, no significant differences were found in arginine conversion which accounted in case of ornithine to 2.48±1.08 µmol/g protein in tissues of C mice and 2.61±1.66 µmol/g protein in tissues of HF mice and in case of citrulline to 1.34±0.48 µmol/g protein in tissues of C mice and 1.59±0.80 µmol/g protein in tissues of HF mice (ornithine: *p* = 0.762, citrulline: *p* = 0.206).

Citrulline produced in the intestine can be reconverted to arginine in the kidney. We therefore also determined the amino acid levels in intestine (data provided in Table S3) and kidney (data provided in Table S4) in study 2 but no differences in the tissue levels of citrulline, ornithine and arginine in these two organs were observed. Only the levels of argininosuccinate were found to be reduced in the small intestine (Table 3).

**Table 3:** Fold-changes of selected amino acids in small intestine and kidney in DIO mice after 12 weeks of HF feeding.

	Small intestine		Kidney	
Amino acid	FC	*p*-value	FC	*p*-value
**Citrulline**	1.21	0.091	1.14	0.207
**Ornithine**	1.09	0.317	1.19	0.276
**Arginine**	0.99	0.963	0.93	0.420
**Argininosuccinate**	0.61	0.023	ND	-
**Sum**	0.98	0.795	0.93	0.417

Amino acids of small intestine and kidney were measured via LC-MS/MS after 12 weeks dietary intervention, when mice were either fed a C diet (11 energy% of fat, n = 9) or a HF diet (60 energy% of fat, n = 8) in study 2.

FC: fold-change (HF group/C group). *P-*value obtained by unpaired Student's t-test. Abbreviation: >ND, not detectable.

### Effect of HF feeding on hepatic and skeletal muscle amino acids

**Figure 3:**
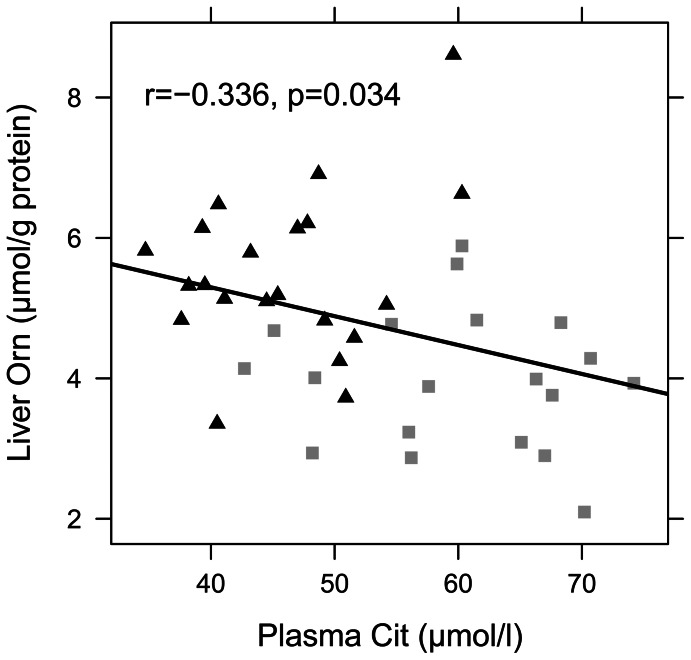
Pearson correlation between concentrations of plasma citrulline and liver ornithine. Mice were fed either a C (10 energy% or 11 energy% of fat) or a HF (45 energy% or 60 energy% of fat) diet for 12 weeks. Black triangles represent C mice (n = 21), grey squares HF mice (n = 19). Abbreviations: Cit, citrulline; Orn, ornithine.

**Figure 4:**
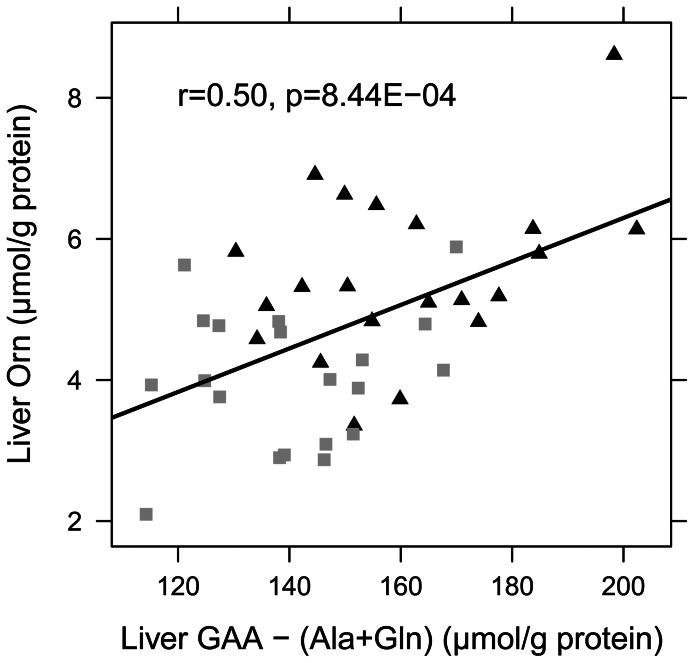
Pearson correlation between concentrations of liver ‘GAA–(Ala+Gln)’ and liver ornithine. Mice were fed either a C (10 energy% or 11 energy% of fat) or a HF (45 energy% or 60 energy% of fat) diet for 12 weeks. Black triangles represent C mice (n = 21), grey squares HF mice (n = 20). Abbreviations: Ala, alanine; GAA, glucogenic amino acids; Gln, glutamine; Orn, ornithine.

In contrast to kidney and intestine, hepatic amino acid concentrations in both HF feeding trials displayed significant differences in a variety of amino acids. In study 1, 65% of the quantified amino acids were altered significantly, whereas in study 2, 58% of the quantified amino acids changed significantly. Fourteen amino acids displayed similar changes in both feeding trials (data provided in Table S5 and S6). However, the sum of all amino acids in liver remained unchanged (Table 4). Considering the amino acids involved in the urea cycle metabolism, animals on HF diets displayed significantly lower ornithine levels compared to mice on a C diet, while citrulline levels remained unchanged (Table 4). Moreover, Pearson correlation analysis revealed an association between hepatic ornithine levels and plasma citrulline concentrations (Figure 3). Hepatic contents of glucogenic amino acids (GAA) did not differ between the dietary groups in both feeding trials. However, when the high abundant amino acids alanine (Ala) and glutamine (Gln) were omitted, the remaining glucogenic amino acids ‘GAA-(Ala+Gln)’ displayed reduced levels in HF animals compared to C animals (Table 4). These levels again correlated with hepatic ornithine concentrations (Figure 4), which suggests an interrelationship between these amino acids in hepatic metabolism. In addition, DIO mice showed diminished hepatic concentrations of BCAA, AAA, essential amino acids (*p* = 0.051 for study 1) as well as of ketogenic amino acids (Table 4). Analysis of skeletal muscle amino acid levels (data provided in Table S7) revealed that in DIO mice citrulline concentrations were increased (C: 2.55±0.30 µmol/g protein vs. HF: 3.07±0.43 µmol/g protein, *p* = 0.012), while arginine concentrations were reduced (C: 4.86±0.84 µmol/g protein vs. HF: 3.87±0.72 µmol/g protein, *p* = 0.020) and ornithine concentrations remained unchanged (C: 0.86±0.19 µmol/g protein vs. HF: 0.87±0.19 µmol/g protein, *p* = 0.914). These changes however resulted in diminished arginine bioavailability in skeletal muscle (C: 1.43±0.25 vs. HF: 0.99±0.15, *p*<0.001) and a significant association was found between citrulline levels in plasma and skeletal muscle (r = 0.61, *p* = 0.012). Furthermore, in skeletal muscle essential amino acids were reduced (C: 52.65±5.19 µmol/g protein vs. HF: 46.4±5.03 µmol/g protein, *p = *0.024) and ketogenic amino acids also declined significantly (C: 24.24±4.32 µmol/g protein vs. HF: 17.88±2.67 µmol/g protein, *p = *0.003).

**Table 4:** Fold-changes of selected (groups of) amino acids in liver in DIO mice after 12 weeks of HF feeding.

	study 1		study 2	
Amino acid	FC	*p*-value	FC	*p*-value
**Citrulline**	1.30	0.058	0.84	0.128
**Ornithine**	0.74	0.003	0.74	0.004
**Arginine**	ND	-	ND	-
**Glucogenic AA**	1.02	0.670	0.98	0.766
**GAA-(Ala+Gln)**	0.89	0.008	0.85	0.009
**Ketogenic AA**	0.83	0.006	0.80	0.004
**BCAA**	0.86	0.033	0.77	0.003
**Aromatic AA**	0.83	0.019	0.85	0.015
**Essential AA**	0.91	0.051	0.83	0.007
**Sum**	1.06	0.126	1.01	0.820

Mice (n = 12 per group) were either fed a C diet (10 energy% of fat) or a HF diet (45 energy% of fat) in study 1. In study 2, mice received either a C diet (11 energy% of fat, n = 9) or a HF diet (60 energy% of fat, n = 8).

Abbreviations: AA, amino acids; GAA, glucogenic amino acids; Ala, alanine; Gln, glutamine; BCAA, branched-chain amino acids; FC, fold-change (HF group/C group); ND, not detectable. *P-*value obtained by unpaired Student's t-test.

### Plasma amino acids in the insulin-deficient state

To compare the effects of such diet-induced metabolic perturbations with the effects in an insulin-deficient diabetic condition, we used a type 1 diabetes model by treating C57BL/6 mice with STZ. The loss of insulin led to a marked reduction in body weight and an almost 4-fold increase in blood glucose concentration 5 days after STZ treatment (Table 1). Plasma amino acid profiling in these mice revealed a prominent increase of valine, leucine, and isoleucine levels. The aromatic amino acids phenylalanine and tyrosine, as well as the total sum of all measured amino acids also showed elevated levels (Table 2 and Table S8). In addition, the diabetic mice displayed a 3-fold elevated concentration of α-aminobutyrate and increased ornithine and citrulline levels compared to C animals (Table 2). Like for DIO mice, in the insulin-deficient mice systemic arginine bioavailability was diminished compared to C mice (C: 1.01±0.11 vs. STZ: 0.59±0.08, *p*<0.001) and a high correlation was found between plasma ornithine and citrulline levels (r = 0.89, *p*<0.001). Thus, citrulline and ornithine showed similar changes in both models while only in the type 1 diabetes model BCAA, phenylalanine and tyrosine levels increased in plasma.

## Discussion

Various metabolomics approaches in humans have shown increased plasma amino acid levels (mainly BCAA and AAA) in states of obesity, IR and T2DM. Additionally, plasma concentrations of free fatty acids and ketone bodies have as well been identified as strongly associated with the obese and diabetic state [14]–[16], [26], [27]. We here describe that similar changes in plasma amino acids can be observed in a type 1 diabetic mouse model, but not in DIO models that are used to simulate the metabolic syndrome associated with hyperglycemia, hyperinsulinemia and non-alcoholic fatty liver disease.

Although all mice fed a HF diet for 12 weeks displayed hyperglycemia and increased liver triglyceride content, common significant diet-induced changes in plasma amino acid levels were only found for citrulline and ornithine. Both feeding trials employed diets that differed in composition by total fat content and dietary fat source and additionally the nutritional state varied as mice were either fasted (study 1) or non-fasted (study 2) when they were sacrificed. Therefore, the consistent changes found in plasma citrulline and ornithine levels suggest those to arise from the metabolic condition and not from dietary differences. Despite the fact that other changes in plasma amino acids were observed over time, neither plasma BCAA nor phenylalanine levels revealed significant differences between HF and C animals after 12 weeks. The increases found here in plasma citrulline as associated with DIO are however consistent with data reported by Connor *et al.* in leptin receptor-deficient db/db mice [28] and those reported by She *et al.* in leptin-deficient ob/ob mice [18]. When in mice on a HF diet a part of the protein was replaced by ketogenic essential amino acids, animals gained less body weight, had improved blood glucose and insulin concentrations, and displayed lower plasma citrulline levels after 8 weeks of intervention compared to mice on a normal HF diet [29]. This is in support of our findings with a close association between the obesity state and plasma citrulline levels. Like for study 1, a time-dependent raise of plasma citrulline concentration was also shown in AKITA type 1 diabetic mice [19]. In contrast, findings in human studies are far less consistent. While increased plasma citrulline concentrations were reported recently in severely obese subjects with hyperglycemia and were strongly associated with elevated levels of glycosylated hemoglobin [30], other studies reported a small decrease in plasma citrulline or unaltered levels in obese subjects [31] and type 1 diabetic patients [32].

What makes citrulline unique is a distinct inter-organ cycle that is thought to ensure a constant systemic arginine concentration [33]. As citrulline can be derived from arginine via nitric oxide (NO)-synthase, a systemic arginine bioavailability index (ratio of arginine to sum of ornithine and citrulline) is frequently determined as a surrogate of a reduced NO synthesis capacity [23], [24], [34], [35]. In our DIO mice and the type 1 diabetic mice, a diminished systemic arginine bioavailability was consistently found. This finding matches with reports of a reduced arginine bioavailability in apoE^−/−^ mice fed a HF diet and in C57BL/6 mice supplied with a high-cholesterol diet [23]. Humans with type 2 diabetes also display a reduced arginine bioavailability index [34], [35] associated with endothelial dysfunction and the progression of cardiovascular diseases. Systemic arginine is mainly derived from kidneys by re-conversion of citrulline which originates mainly from the intestine. Precursors of intestinal citrulline are either dietary arginine or endogenous glutamine [36]. Systemic increases in citrulline levels as observed in DIO and type 1 diabetic mice could therefore originate from either an increased intestinal production or a reduced conversion of citrulline to arginine in kidney. Our stable isotope study with isolated intestinal tissues revealed that neither ornithine nor citrulline production from luminally supplied arginine was altered in DIO animals. Although this may not relate to the *in vivo* condition, intestinal arginine extraction is known to depend on the total protein supply and increases in states of protein starvation but not in response to over-nutrition [37], [38]. The second source of systemic citrulline is endogenous glutamine. Whether the conversion of glutamine to citrulline in the intestine is increased in the DIO state is currently not known and deserves additional studies. As kidney is the prime tissue for systemic arginine levels, it is not surprising that elevated plasma citrulline levels also occur in states of renal insufficiency and proximal tubular dysfunction as shown in rats [39] and in humans with renal failure [40]. It however appears unlikely that a renal dysfunction may have caused the elevation in plasma citrulline in DIO mice since those changes occurred as early as 2 weeks after feeding the HF diet. Moreover, amino acid profiling of renal and intestinal tissues revealed a reduction of argininosuccinate levels in the intestine, but no differences were found for citrulline, ornithine, or arginine levels in DIO mice in either tissue.

In contrast to kidney and intestine, skeletal muscle displayed an increase in citrulline and a reduction in arginine levels and thus a reduced arginine bioavailability. Although muscle may not be considered as a prime organ in arginine and citrulline metabolism, it has been shown that palmitic acid enhances NO production in rat skeletal muscle cells [41] and that mice lacking the inducible NO-synthase in skeletal muscle are protected from HF induced insulin resistance [42]. With the association between plasma and skeletal muscle citrulline levels found here in DIO mice, it is tempting to speculate that muscle citrulline could also contribute to the observed plasma increases.

Citrulline is also a product of the urea cycle in liver. Although animal experiments [36] suggested that liver does not extract systemic citrulline, human studies have demonstrated that there is a significant net uptake of circulating citrulline into hepatocytes [43]. This finding is in contrast to stable isotope tracer studies in rat liver that demonstrated even a hepatic citrulline release from labeled glutamine as a precursor [44]. When liver samples in our DIO mice were profiled for changes in amino acids, significant alterations in a variety of amino acids including ornithine but not citrulline were observed. Arginine was below detection limits. Interestingly, in both HF feeding trials liver ornithine levels correlated with plasma citrulline levels. Moreover, liver ornithine status correlated with the levels of glucogenic amino acids, but only when alanine and glutamine were not taken into account. These two amino acids are of course important precursors of hepatic *de novo* glucose production but their high levels appear to mask underlying alterations in other glucogenic amino acids that link to changes in ornithine concentrations. HF feeding in mice and rats has been shown to enhance hepatic glucose production [45], [46] and the reduced levels of glucogenic amino acids other than glutamine and alanine may therefore be indicative for an increased utilization for gluconeogenesis. The inverse relationship found between hepatic ornithine and systemic citrulline levels is of course intriguing. During the urea cycle ornithine has to pass from the cytoplasm, where it is formed, to the mitochondrial matrix, where it is carbamylated. The resulting citrulline must then pass from the matrix to the cytoplasm before being converted to arginine. This bidirectional transport seems to be mediated by an ornithine/citrulline exchanger which is coupled to proton movement to balance charge differences between the substrates [47]. There is evidence that ornithine transport into and release from hepatocytes involves a low affinity, high capacity transport system [48], whereas the transporter(s) responsible for hepatic citrulline uptake and release are essentially unknown. In rat aortic smooth muscle cells, Na^+^-dependent and Na^+^-independent citrulline transport systems could be shown, suggesting systems N and L like activity [49]. In contrast to the system L-transporter LAT1 which seems to be positively regulated by insulin [50], the expression of the Na^+^-dependent system N transporter SNAT3 is reduced when mouse hepatocytes were chronically treated with insulin [51]. The increase in plasma citrulline levels in DIO mice could therefore arise from a reduction in SNAT3 transport activity although its involvement in hepatic citrulline transport remains to be determined. That the changes in systemic citrulline levels are related to insulin action is supported by the observed increase in plasma citrulline and ornithine levels also in the type 1 diabetic mice. As liver tissues in DIO models show impaired insulin responses, the changes in plasma citrulline and ornithine may serve as a surrogate marker of this hepatic perturbation.

In contrast to the DIO mice, type 1 diabetic mice displayed significantly increased plasma BCAA levels and tyrosine and phenylalanine were elevated as well. The changes in BCAA and citrulline levels are very similar to those reported in AKITA mice as another type 1 diabetes model [19] and mimic the findings in diabetic humans [15], [52]. What causes these changes in the BCAA and AAA in diabetic states is currently not understood. The prime organ in BCAA catabolism is the skeletal muscle and under fasting conditions the release of BCAA and other amino acids from muscle is increased [53]. There is now growing evidence that IR and a diabetic state display plasma metabolome changes reminiscent of those observed in healthy volunteers under extended fasting conditions [54]. Fasting causes increased rates of lipolysis and proteolysis with concomitant elevations of plasma free fatty acids and amino acids. The almost 3-fold increase in α-aminobutyrate levels in type 1 diabetic mice may also serve as an indicator of the increased protein breakdown and amino acid utilization in insulin-deficiency as α-aminobutyrate is a product of threonine and methionine catabolism [55], [56]. Of note, the most discriminative marker of a diabetic state in humans found so far is 2-OH-butyrate which derives from the same metabolic pathways [26].

In summary, we demonstrate increased plasma citrulline levels when mice progress into an obese state associated with hyperglycemia and development of non-alcoholic fatty liver disease. These changes translate into a decreased systemic arginine bioavailability that may affect the capacity to produce NO. Tissue profiling revealed major changes in hepatic amino acid levels in DIO mice. Changes in selected liver amino acids but also skeletal muscle arginine and citrulline levels could be correlated with elevated plasma citrulline levels. Type 1 diabetic mice showed similar increases in plasma citrulline and ornithine and reduced arginine bioavailability as DIO mice, but in addition they displayed all other alterations in plasma amino acids reported for humans with IR and diabetes such as elevated BCAA, tyrosine, and phenylalanine levels. Despite the fact that DIO C57BL/6 mice do not produce the characteristic plasma amino acid changes as in humans with IR and diabetes, increases of plasma citrulline follow closely obesity development and may therefore be predictive for the metabolic syndrome.

## Materials and Methods

### Ethic statements and mouse studies

#### HF Study 1

As described previously [21], [22], this study was part of the Proof of principle package of the European Nutrigenomics Organisation. All procedures were performed in accordance with the recommendations in the Guide for the Care and Use of Laboratory Animals at Wageningen University. The study was approved by the Ethical Committee on animal testing of Wageningen University (Study number 2007118.b). At the age of 12 weeks, 24 male C57BL/6J mice were fed a low-fat diet (10% of energy obtained from fat) as a run-in period for four weeks. The mice were then divided into two groups (n = 12) and maintained either on the C (low-fat) diet or received a HF diet (45% of energy obtained from fat). Diets contained palm oil as the main fat source and were based upon Research Diets formulas (www.researchdiets.com; D12450B/D12451). Throughout the feeding trial the mice had *ad libitum* access to food and water. Body weight and food intake were monitored weekly [22]. Every second week, plasma was collected into EDTA-coated tubes by puncture of the retro-orbital sinus. After twelve weeks, the mice were fasted for five hours, followed by plasma collection under anaesthesia using a mixture of isoflurane (1.5%), nitrous oxide (70%), and oxygen (30%). Mice were then sacrificed by cervical dislocation and the liver was sampled for amino acid analysis. Samples were frozen in liquid nitrogen and stored at −80°C. Blood glucose levels were measured after sacrifice using an Accu-Check glucose meter (Roche Diagnostics, Almere, The Netherlands) [22].

#### HF study 2

This study was performed in C57BL/6N mice. All procedures were conducted according to the German guidelines for animal care and approved by the state of Bavaria (Regierung von Oberbayern) ethics committee (Reference number 209.1/211-2531-41/03). Animals were purchased from Charles River Laboratories at the age of eight weeks and maintained at a 12 h light/dark cycle with *ad libitum* access to food and water. At the age of ten weeks, the mice were randomized into two groups (n = 9): a C diet (11% of energy obtained from fat, Ssniff GmbH, Soest, Germany; E15000-04) or a HF diet (60% of energy obtained from fat, Ssniff GmbH, Soest, Germany; E15741-34) group. After 11 weeks of dietary intervention, mice were fasted for 6 hours and blood glucose was measured using a blood spot of the tail vein and the Accu-Check glucose meter. After 12 weeks, non-fasted mice were anaesthetized using isoflurane and blood was collected by puncture of the retro-orbital sinus into lithium heparin coated tubes. After sacrificing the mice by cervical dislocation, the small intestine was removed and used for gut sac experiments as described below. For amino acid analysis liver, small intestine, kidney, and skeletal muscle tissues were snap frozen in liquid nitrogen and stored at −80°C.

The nutrient compositions of the diets used in the two feeding trials are displayed in Table 5.

**Table 5:** Nutrient composition of experimental diets used for the HF feeding trials.

	study 1		study 2	
	C	HF	C	HF
**Crude protein**	200	200	208	241
**Crude fat**	45	202.5	42	340
**Beef tallow**	-	-	-	310
**Soybean oil**	25	25	40	30
**Palm oil**	20	177.5	-	-
**Crude fiber**	50	50	50	50
**Starch**	427.5	72.8	468	22
**Sugar**	172.8	172.8	108	224
**Maltodextrine**	100	100	-	-
**Dietary energy-% carbohydrate**	69	35	66	21
**Dietary energy-% protein**	20	20	23	19
**Dietary energy-% fat**	10	45	11	60

Nutrient composition expressed as (g/kg) except not stated otherwise.

#### Type 1 diabetic mice

Twelve weeks old male C57BL/6J mice were maintained at 12∶12 h light/dark cycle with food and water *ad libitum*. Mice received a standard laboratory chow diet (Ssniff GmbH, Soest, Germany; V1534) and mice were kept in pairs. All experiments were conducted according to the German guidelines for animal care and approved by the state of Bavaria (Regierung von Oberbayern) ethics committee (Reference number: 55.2.1-54-2532-22-11). The STZ-treated group (n = 10) received a single intraperitoneal dose of 180 mg/kg body weight STZ (Sigma Aldrich, USA) in 0.1 M citric acid buffer (pH = 4.5). C mice (n = 10) were injected intraperitoneally with 0.1 M citric acid buffer (pH = 4.5) alone. After 5 days, blood glucose was measured using a blood spot of the tail vein and the Accu-Check glucose meter. Afterwards, mice were anaesthetized using isoflurane and plasma was collected by orbital puncture into EDTA-coated tubes.

### Amino acid analysis

For quantification of amino acids the targeted LC-MS/MS approach with iTRAQ® (AA45/32™ Phys REAG Kit, Applied Biosystems, USA) was used as described previously [57]. For measurement of tissue amino acids and derivatives (skeletal muscle, liver, small intestine, kidney) small pieces of tissues were homogenized in liquid nitrogen. 100 mg of grounded tissue were dissolved in 150 µl MeOH/H_2_O (50∶50 v/v), vortexed, and centrifuged. Sample preparation was done according to the manufacturer's instructions using 40 µl of the supernatant of the tissue homogenates or 40 µl plasma. Tissue amino acid concentrations were standardized with protein concentrations determined via Bradford assay (Bio-Rad Laboratories GmbH, Germany). Plasma amino acids of the STZ-trial were determined after derivatization with aTRAQ® (aTRAQ™ Reagent Kit, ABSciex, Foster City, USA). Sample preparation was done according to the manufacturer's instructions using 40 µl plasma.

### Everted gut sacs

To determine the capacity of the intestine to convert arginine to citrulline *in vitro* we used ^13^C^15^N-labeled arginine (labeling rate for ^13^C: 98%, and for ^15^N: 98%; Silantes GmbH, Germany) as a substrate. The use of ^13^C^15^N-arginine (M = 184.1 g/mol) enabled to distinguish between citrulline (M = 182.1 g/mol) produced via ornithine and citrulline (M = 184.1 g/mol) produced by nitric oxide (NO) synthase. The whole small intestine was taken out, mesenteric fat as well as the proximal 7 cm of small intestine were removed. The tissue was then washed and rinsed with ice-cold Krebs buffer (119 mM NaCl, 4.7 mM KCl, 2.5 mM CaCl_2_, 1.2 mM MgSO_4_, 1.2 mM KH_2_PO_4_, 25 mM NaHCO_3,_ and 1% HEPES, pH 7.4) and everted. The intestine was cut into 2 cm segments beginning at the proximal site. Per mouse, 2 to 4 sacs were prepared and filled with Krebs buffer. Sacs were directly incubated at 37°C for 30 min on a shaker in 2 ml Krebs buffer solution containing 0.1 mM ^13^C^15^N-labeled arginine and 2 mM glutamine, as glutamine was shown to enhance intestinal citrulline production [58]. During the incubation Krebs buffer was oxygenated with carbogen (5% CO_2_/95% O_2_). After incubation tissues were washed with ice-cold Krebs buffer and serosal and mucosal fluids were collected and stored at −80°C. The concentrations of ^13^C^15^N-labeled arginine and its products citrulline and ornithine were determined by iTRAQ-labeling using 40 µl of the collected fluids. Intestinal ornithine and citrulline release were standardized with protein concentrations which were determined by Bradford assay after sacs were dried over-night at and digested using 1 M NaOH at 37°C.

### Statistical analysis

Results are presented as mean ± SD or as fold-changes. Statistical analysis was performed using the R software package 2.12 [59]. Tests for statistical significance were either done by two-way ANOVA (diet×time interaction for HF study 1) following pair-wise comparison with Tukey post-hoc test or by unpaired Student's t-test. Significance was assumed when *p*<0.05. Correlation analysis was performed within the *Hmisc* package using the function *rcorr* and Pearson method.

## Supporting Information

Table S1 **Plasma amino acid concentrations of study 1 over the time-course of 12 weeks HF feeding.** Mice were fed a C diet (10 energy% from fat, n = 12) or HF diet (45 energy% from fat, n = 12) for 12 weeks and plasma was analyzed every second week. Data displays all analyzed amino acids plus sum of all amino acids.
(XLS)

Table S2 **Plasma amino acid concentrations of study 2 after 12 weeks of HF feeding.** Mice were fed a C diet (11 energy% from fat, n = 9) or HF diet (60 energy% from fat, n = 7) for 12 weeks. Data displays all analyzed amino acids plus sum of all amino acids.
(XLS)

Table S3 **Amino acid concentrations of the small intestine after 12 weeks of HF feeding.** Mice were fed a C diet (11 energy% from fat, n = 9) or HF diet (60 energy% from fat, n = 8) for 12 weeks. Data displays all analyzed amino acids plus sum of all amino acids.
(XLS)

Table S4 **Renal amino acid concentrations after 12 weeks of HF feeding.** Mice were fed a C diet (11 energy% from fat, n = 9) or HF diet (60 energy% from fat, n = 8) for 12 weeks. Data displays all analyzed amino acids plus sum of all amino acids.
(XLS)

Table S5 **Hepatic amino acid concentrations of study 1 after 12 weeks of HF feeding.** Mice were fed a C diet (10 energy% from fat, n = 12) or HF diet (45 energy% from fat, n = 12). Data displays all analyzed amino acids plus sum of all amino acids.
(XLS)

Table S6 **Hepatic amino acid concentrations of study 2 after 12 weeks of HF feeding.** Mice were fed a C diet (11 energy% from fat, n = 9) or HF diet (60 energy% from fat, n = 8) for 12 weeks. Data displays all analyzed amino acids plus sum of all amino acids.
(XLS)

Table S7 **Skeletal muscle amino acid concentrations after 12 weeks of HF feeding.** Mice were fed a C diet (11 energy% from fat, n = 9) or HF diet (60 energy% from fat, n = 8) for 12 weeks. Data displays all analyzed amino acids plus sum of all amino acids.
(XLS)

Table S8 **Plasma amino acid concentrations of mice 5 days after STZ treatment.** Mice received either a single intraperitoneal injection of streptozotocin (STZ) (180 mg/kg bodyweight) in 0.1 M citrate buffer or 0.1 M citrate buffer alone (control group). Amino acids were assessed 5 days after injection. Data displays all analyzed amino acids plus sum of all amino acids.
(XLS)
